# Genetic diversity of different breeds of Kazakh sheep using microsatellite analysis

**DOI:** 10.5194/aab-62-305-2019

**Published:** 2019-06-05

**Authors:** Kairat Dossybayev, Zarina Orazymbetova, Aizhan Mussayeva, Naruya Saitou, Rakhymbek Zhapbasov, Bolathan Makhatov, Bakytzhan Bekmanov

**Affiliations:** 1 Laboratory of Animal Genetics and Cytogenetics, Institute of General Genetics and Cytology, Almaty, Kazakhstan; 2 Population Genetics Laboratory, National Institute of Genetics, Mishima, Japan; 3 Faculty of Bioresources and Technology, Kazakh National Agrarian University, Almaty, Kazakhstan; 4 Faculty of Biology and Biotechnology, Al-Farabi Kazakh National University, Almaty, Kazakhstan

## Abstract

A total of 75 individuals from five sheep populations in Kazakhstan were investigated based on 12 STR (short tandem repeat, also known as microsatellite) markers in order to study their genetic structure and phylogenetic relationship based on genetic distances. These sheep had a high level of genetic diversity. In total, 163 alleles were found in all the populations using 12 microsatellite loci. The mean number of alleles, effective number of alleles, and polymorphism information content (PIC) values per loci were 13.4, 5.9, and 0.78, respectively. Comparing the allelic diversity between the populations, the highest genetic diversity was observed in the Edilbay-1 sheep breed (8.333±0.644), and the lowest parameter was for Kazakh Arkhar-Merino (7.083±0.633). In all populations, there is a deficiency of heterozygosity. The largest genetic diversity was found in loci *INRA023* and *CSRD247* with 16 alleles, and the smallest polymorphism was noted for the locus *D5S2* with 8 alleles. The level of observed heterozygosity was in the range 0.678±0.051 for Kazakh Arkhar-Merino and 0.767±0.047 for Kazakh fat-tailed coarse wool. The expected heterozygosity level range was from 0.702±0.033 for Kazakh Arkhar-Merino to 0.777±0.023 for Edilbay-1. When 12 microsatellite loci are compared, the *OarFCB20* locus showed the highest level of genetic variability. Excess of heterozygosity was observed at three loci; *MAF065*, *McM042*, and *OarFCB20*. The highest genetic distance was observed between Kazakh Arkhar-Merino and Edilbay-1, whereas the genetic distance between Edilbay-1 and Edilbay-2 is the smallest using Nei's standard genetic distance. The Edilbay-1 sheep breed possesses the largest genetic diversity among these five populations.

## 1 Introduction

Sheep breeding is the most ancient branch of animal husbandry in Kazakhstan. The country has more than 20 distinct sheep breeds to date. Among them, the sheep breeds which first appeared in Kazakhstan by origin and history are Edilbay, Kazakh Arkhar-Merino, Kazakh Finewool, and Kazakh fat-tailed coarse wool. These breeds are well adapted to the various climatic conditions in Kazakhstan. Various breeds of sheep in one way or another are different in terms of biological efficiency. This study aims at investigating the genetic diversity of local sheep breeds. The animals studied belong to purebred and farms' own selection and tribal cards. The Kazakh Arkhar-Merino sheep breed was investigated on Kumtekey breeding farm, where the best sheep of this breed are kept. The Kazakh Arkhar-Merino is based on the interspecific hybridization of wild Arkhar rams with fine wool ewes of Novo-Caucasian Merino, Précoce, and Rambouillet breeds that occurred during 1934–1950. R-Kurty farm is also a breeding farm where the highly productive animals of the Kazakh Finewool breed are concentrated. Edilbay sheep breed is bred in Birlik breeding centre, where the purebred and highly productive sheep of this breed are kept. The Kazakh fat-tailed coarse wool sheep breed is the product of popular selection which has lasted many years. They are widely bred in many sheep farms all over Kazakhstan. However, highly productive animals typical of this breed are concentrated in Kabyl-Nur farm. Thus, four sheep breeds selected by us for molecular genetic studies are, firstly, the most common sheep breeds in Kazakhstan, which are well adapted to different climatic conditions of breeding and housing. Secondly, these breeds differ from each other in their origin and method of breeding, and thus they are excellent subjects for comparative molecular genetics research. Thirdly, the specification of breeding farms from which sheep were studied is extremely important, since we use the results of the molecular genetics study of these breeds in these farms to improve breeding work and speed up the selection process in order to create a highly productive breeding core in a short period of time. Since research started in 2010, the molecular database has grown, and the characterization of genetic diversity in farm animals has become particularly pertinent (Groeneveld et al., 2010).

Today, the FAO and the ISAG–FAO Advisory Group on Animal Genetic Diversity recommend specific sets of STR (short tandem repeat, also known as microsatellite) loci for genetic analyses, such as for horse, cattle, and pig breeds. Of the different types of molecular markers, STRs are suitable for studying genetic diversity because of their abundance – the large amount of allelic variation at each locus is highly polymorphic, their distribution throughout the genome is random, and inheritance is codominant (Rekha et al., 2016; Barcaccia et al., 2013; Putman et al., 2014). In addition, STRs are able to generate information for the planning of crossings and further selection of genotypes in genetic breeding programs (Faleiro et al., 2007; Crispim et al., 2014). The objective of the current research was to investigate 12 STR loci based on genetic diversity in sheep herds as well as the differentiation and relationship among the number of alleles and genetic links between Kazakh sheep breeds.

## 2 Materials and methods

Blood samples were taken from five sheep populations: two of them were Edilbay-1, though of two various Edilbay-1 sheep herds, and three others were different sheep breeds in the categories Kazakh Finewool, Kazakh Arkhar-Merino, and Kazakh fat-tailed coarse wool. The 15 animals were chosen randomly from each population. Genomic DNA was extracted using a commercial kit (GeneJET Genomic DNA Purification Kit, ThermoFisher Scientific, USA). Both the quality and concentration of DNA were verified by spectrophotometric and agarose gel electrophoresis. In this study, 12 STR primers were used, and all of them are recommended by the International Society of Animal Genetics (ISAG, 2017). Amplification was carried out using a Tetrad 2 thermal cycler (Bio-Rad). Polymerase chain reaction (PCR) products were attached in the ABI310 Genetic Analyser, and GeneMapper software was used to determine fragment size. The number of alleles, effective number of alleles, polymorphism information content (PIC) values, observed and expected heterozygosities, Wright's F statistics per locus, pairwise population $F_{\mathrm{st}}$ values, and pairwise population matrix of Nei's standard genetic distance were calculated using GenAlex 6.5 and Excel microsatellite toolkit (version 3.1) software (Peakall and Smouse, 2012; Park 2008). The neighbour joining method of Saitou and Nei (1987) was used to construct a phylogenetic tree based on Nei's genetic distance in MEGA7 (Kumar et al., 2016). Factorial correspondence analysis (FCA) was investigated based on the individual multi-locus genotype using GENETIX version 4.03 (Belkhir et al., 1996).

## 3 Results

All examined markers were polymorphic for all the populations examined. In total, 161 alleles were found in five native Kazakh sheep breeds based on 12 STR loci (Table 1). The highest number of alleles was 16 for markers *INRA023* and *CSRD247*, whereas *D5S2* showed the lowest number (8 alleles), and the average number of alleles was 13.4 per locus.

**Table 1:** Genetic diversity analysis of sheep populations based on the 12 microsatellite markers. Number of alleles ($N_{a}$), effective number of alleles ($N_{e}$), observed heterozygosity ($H_{o}$), expected heterozygosity ($H_{e}$), polymorphism information content (PIC), and F statistics ($F_{\mathrm{is}}$, $F_{\mathrm{it}}$, and $F_{\mathrm{st}}$).

Locus	$N_{a}$	$N_{e}$	$H_{o}$	$H_{e}$	PIC	$F_{\mathrm{is}}$	$F_{\mathrm{it}}$	$F_{\mathrm{st}}$
*CSRD247*	16	7.862	0.720	0.873	0.8566	0.131	0.175	0.051
*D5S2*	8	3.654	0.667	0.726	0.6966	0.032	0.082	0.052
*INRA005*	14	7.263	0.787	0.862	0.8376	0.041	0.088	0.049
*INRA006*	12	4.257	0.520	0.765	0.7194	0.230	0.320	0.117
*INRA023*	16	9.007	0.813	0.889	0.8654	0.022	0.085	0.064
*INRA63*	14	8.152	0.840	0.877	0.8535	-0.030	0.043	0.070
*INRA172*	15	3.733	0.573	0.732	0.7126	0.157	0.217	0.071
*MAF065*	13	4.643	0.813	0.785	0.7547	-0.072	-0.037	0.033
*MAF214*	15	4.568	0.653	0.781	0.7318	0.068	0.164	0.103
*McM042*	12	3.216	0.733	0.689	0.6496	-0.115	-0.064	0.045
*McM527*	13	5.890	0.773	0.830	0.8054	-0.007	0.069	0.075
*OarFCB20*	13	9.131	0.893	0.890	0.8766	-0.097	-0.003	0.085
Mean	13.416	5.948	0.732	0.808	0.78	0.030	0.095	0.068

The effective number of alleles for each marker varied between 3.2 and 9.1, with a mean value of 5.9. The PIC values ranged from 0.65 to 0.88, with a mean of 0.78. Observed heterozygosity values varied from 0.52 to 0.89, with an average of 0.73, while the expected heterozygosity values ranged from 0.69 to 0.89 with a mean value of 0.81 (Table 1). Our study demonstrated a high genetic polymorphism in the investigated sheep breeds. These estimates are higher than those reported for other sheep breeds (Ferrando et al., 2014, in France and Spain; Salamon et al., 2014, in Croatia and Bosnia and Herzegovina; Al-Atiyat et al., 2015, in Australia; Gaouar et al., 2016, in Morocco).

**Table 2:** Genetic diversity within the five sheep populations. Mean number of alleles (MNA), effective number of alleles ($N_{e}$), observed heterozygosity ($H_{o}$), and expected heterozygosity ($H_{e}$).

Population	MNA	$N_{e}$	Pa	$H_{o}$	$H_{e}$
Kazakh Arkhar-Merino	7.083±0.633	3.902±0.477	16	0.678±0.051	0.702±0.033
Kazakh Finewool	7.917±0.557	4.900±0.538	14	0.744±0.048	0.770±0.022
Edilbay-1	8.333±0.644	4.975±0.461	13	0.739±0.052	0.777±0.023
Edilbay-2	7.583±0.417	4.343±0.373	6	0.733±0.034	0.750±0.022
Kazakh fat-tailed coarse wool	7.417±0.609	4.921±0.524	6	0.767±0.047	0.768±0.025
Total	7.667±0.256	4.608±0.214	55	0.732±0.021	0.753±0.012

The average number of alleles, the effective number of alleles, and the expected and observed heterozygosity for each breed are shown in Table 2. The average number of alleles for the Edilbay-1 breed is 8.33. This analysis showed that there was no significant differentiation among the groups from the following populations: Kazakh Arkhar-Merino (7.08), Kazakh Finewool (7.91), Edilbay-2 (7.58), and Kazakh fat-tailed coarse wool (7.41). With the exception of $N_{e}=3.90$ in Kazakh Arkhar-Merino sheep, the effective number of alleles was larger than 4.0. In this study, the average value of observed heterozygosity was 0.68 for the Kazakh Arkhar-Merino population, and similar results were reported for Chinese and Mongolian breeds (Zhong et al., 2011). It is important to note that this is lower compared to the other four investigated sheep populations. At the same time, the expected heterozygosity value was more than 0.7 for all sheep groups. Values of the comparable means have been reported by Yilmaz et al. (2014).

**Figure 1:**
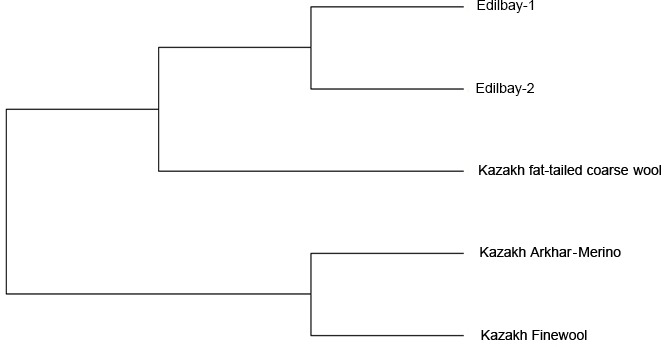
The phylogenetic tree constructed from Nei's standard genetic distances among five sheep populations.

The highest $F_{\mathrm{is}}$ value was observed in marker *INRA006*, while the lowest $F_{\mathrm{is}}$ was recorded for locus *McM527*. The maximum and minimum $F_{\mathrm{it}}$ values were found in markers *INRA006* and *OarFCB20* respectively. $F_{\mathrm{st}}$ values ranged from 0.117 to 0.033, and the average values of $F_{\mathrm{is}}$, $F_{\mathrm{it}}$, and $F_{\mathrm{st}}$ were 0.030, 0.095, and 0.068 accordingly. According to the observed results of $F_{\mathrm{is}}$, related mating occurs within each studied breed ($F_{\mathrm{is}}\;>\;0$). Obtained $F_{\mathrm{st}}$ values showed that the degree of genetic divergence is moderate between the sheep populations ($F_{\mathrm{st}}\;>\;0.06$). The $F_{\mathrm{it}}$ value was higher than zero, which indicates deficiency of heterozygosity. The highest genetic distance was found between Kazakh Arkhar-Merino and Edilbay-1 (0.469), while the smallest genetic distance was observed between Edilbay-1 and Edilbay-2 (0.217). Pairwise values of genetic differentiation, $F_{\mathrm{st}}$, varied from 0.030 to 0.063.

**Figure 2:**
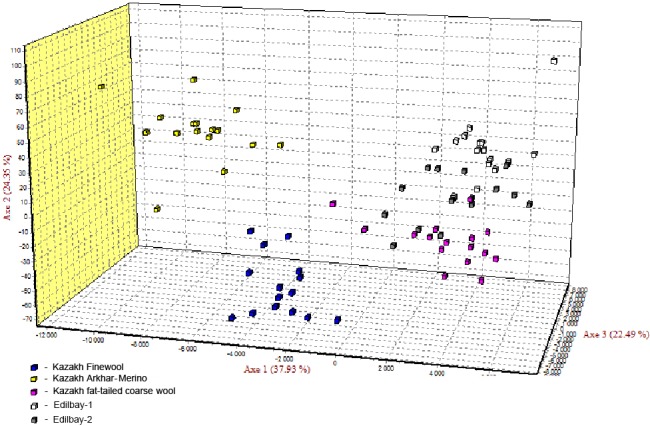
The factorial correspondence analysis of five sheep populations studied on the bases of 12 STR loci.

The neighbour joining tree for all samples was constructed using pairwise population matrix of Nei's genetic distances in order to represent the relationships between the sheep breeds (Fig. 1). Edilbay-1 and Edilbay-2 were initially classified as sub-clusters, which were further clustered into Kazakh fat-tailed coarse wool, whereas Kazakh Arkhar-Merino and Kazakh Finewool sheep breeds were grouped around the same node. Moreover, in the factorial correspondence analysis, the distinction of three clusters is illustrated by three axes showing variances of 37.93 %, 24.35 %, and 22.49 %, respectively (Fig. 2).

## 4 Discussion

In this study, 12 STR (microsatellite) markers were used to evaluate genetic diversity in five populations. The analysis revealed no significant differences in the main genetic characteristics of the interbred population: number of alleles, effective number of alleles, and expected and observed heterozygosities. A high level of genetic diversity was observed in loci *INRA023* and *CSRD247*, with a large number of alleles. According to Ozerov et al. (2008), the Edilbay sheep breed had the highest genetic diversity among the populations studied. Similarly, our analysis of the data showed that the greatest genetic diversity was detected in the Edilbay-1 sheep breed, with a mean value of alleles of 8.333±0.644, and these indicators did not differ much among the remaining populations (Table 2). The allele diversity for Edilbay-1 was similar to that of Arabian sheep studied with 12 microsatellite loci (Ahmed et al., 2018) but much higher than that reported for Jordan sheep breeds (Khaleel et al., 2018). The lowest genetic diversity among the breeds studied was expressed in Kazakh Arkhar-Merino, with an average value of seven alleles. The obtained allele diversity for Kazakh Arkhar-Merino was higher than the other studies (Sadeghi, 2018 and Khaleel et al., 2018), and this indicator is close to that described for the Algerian breeds (Gaouar et al., 2015). Moreover, in the present study the highest effective number of alleles was found for Edilbay-1($N_{e}=4.9$), similar to values reported for Nellore sheep (Vani et al., 2017). The reason why the Edilbay-1 sheep breed maintains high genetic diversity is that currently in Birlik breeding centre there are 16 512 breeding sheep of the Edilbay breed, including 7500 ewes. In addition, farmers raise the sheep in different flocks by dividing them into three different groups according to the colour of their wool. Researchers have shown that animals with different colour wool are characterized by unequal productivity. For example, it has been proven that ewes with black wool have a higher wool yield by 7.5 %–11.8 %, a higher live weight by 2.2 %–6.9 %, and better slaughter qualities than sheep with red wool. The sheep with brown wool are characterized by the same high productivity indices. The fertility of females is not higher than 110 %–120 %. The milk content of the sheep is quite high. Furthermore, during the season of breeding, breeders exchange rams among flocks to maintain the genetic diversity.

**Table 3:** Pairwise population matrix of Nei's genetic distances (above the diagonal) and pairwise population $F_{\mathrm{st}}$ Values (below the diagonal).

	Kazakh Arkhar-	Kazakh Finewool	Edilbay-1	Edilbay-2	Kazakh fat-tailed
	Merino				coarse wool
Kazakh Arkhar-Merino	0.000	0.259	0.469	0.406	0.403
Kazakh Finewool	0.040	0.000	0.401	0.299	0.242
Edilbay-1	0.063	0.046	0.000	0.217	0.328
Edilbay-2	0.059	0.039	0.030	0.000	0.222
Kazakh fat-tailed coarse wool	0.057	0.031	0.039	0.030	0.000

The overall average of *PIC* value for the five populations was equal to 0.78, and all the markers were higher than 0.5, which means all the investigated STR loci were highly informative. This value was higher than that reported for Chinese sheep (PIC of 0.64; Guang-Xin et al., 2016). Also, PIC values showed that the most informative markers were *OarFCB20 *and *INRA023*. However, PIC values were lower than heterozygosities. According to Botstein et al. (1980), PIC must be always less than the expected heterozygosity. Aside from Kazakh Arkhar-Merino, in all examined populations, the average PIC value had a similar ranking. Consequently, these markers are suitable for studying the genetic diversity in Kazakh sheep breeds.

The fixation index was estimated on a per locus basis, and due to negative assortative mating, an excess of heterozygosity was found at markers *MAF065*, *McM042*, and *OarFCB20.*

In addition, to estimate the genetic variability of the studied sheep breeds, we calculated the expected and observed heterozygosity values. Except for *MAF065* and *McM042*, in all loci the level of observed heterozygosity was lower than expected heterozygosity; therefore there were more homozygous individuals in the investigated flocks. As well as the mean expected heterozygosity in each population being higher than that of the heterozygotes, our results showed that heterozygotic deficiency was noted in all populations. Compared to the average heterozygosity value in the population, the observed heterozygotes fluctuated from 0.678±0.051 (Kazakh Arkhar-Merino) to 0.767±0.047 (Kazakh fat-tailed coarse wool), while the expected heterozygotes ranged from 0.702±0.033 (Kazakh Arkhar-Merino) to 0.777±0.023 (Edilbay-1). Meanwhile, the overall average values for this population were 0.732±0.021 and 0.753±0.012 (Table 2). These findings were similar to those reported in the literature (Sassi-Zaidy et al., 2014). The genetic variability obtained in these studies was similar to the results reached by Ozerov et al. (2008). By contrast, the level of genetic variability of the Kazakh Arkhar-Merino sheep breed is lower than those of Edilbay and Kazakh Finewool. In spite of this, this indicator of Kazakh Arkhar-Merino was still higher than those of some native Chinese sheep breeds (Guang-Xin et al., 2016) as well as eastern Adriatic and western Dinaric native sheep breeds (Salamon et al., 2014). The average mean of the observed heterozygosity was less than the mean of expected heterozygosity in the whole population; this could be due to selection against heterozygosity or inbreeding (Abdullah et al., 2013; Carmen et al., 2007). Thus, according to the comparison results of the investigated populations, the genetic variability in the Kazakh Arkhar-Merino population was identified as being lower than in the others.

Previously, a study was conducted by Ozerov et al. (2008) on four sheep breeds of Kazakhstan (Degeres Mutton-wool, Kazakh Arkhar-Merino, Kazakh Finewool, and Edilbaev) using 20 microsatellite loci. As a result, it was determined that all studied sheep breeds showed a high level of polymorphism in all 20 microsatellite loci and were in a state of genetic equilibrium according to the Hardy–Weinberg ratio. However, in our studies of the Kazakh Arkhar-Merino sheep breed, there were significant differences between the expected and observed heterozygosity ($H_{o}=0.678$ and $H_{e}=0.702$). In Ozerov (2008) et al., this index was in equilibrium ($H_{o}=0.72$ and $H_{e}=0.71$). This means that in the Kazakh Arkhar-Merino sheep breed population there is a deficiency of heterozygotes. This fact evidences that the degree of inbreeding of the Kazakh Arkhar-Merino sheep breed is quite high. In the current conditions of animal breeding, when private farm sheep breeding is widely practised in the country, breeding farms for one breed each have a small number of animals. Therefore, they practically do not exchange tribal animals among themselves. However, with a closed breeding system, inbreeding sooner or later reaches a dangerous level and leads to inbreeding depression (decreased productivity and reproductive qualities) and, finally, degeneration. Consequently, it is necessary to strive to limit inbreeding in conserved breeds and gene pool herds. The same situation exists for sheep of the Kazakh Arkhar-Merino breed from Kumtekey breeding farm. Based on the obtained genetic information on a high degree of inbreeding, animals from this farm will develop special breeding measures to preserve the valuable interspecific gene pool of animals of this unique breed and their rational use. We gave recommendations to Kumtekey breeding farm to reduce inbreeding urgently while maximizing the number of males used and reducing the number of lines with the highest intensity of interlinear pairing.

Furthermore, in order to analyse population differentiation and structure, Wright's F statistics of three indices for the overall population were used for each locus (Wright, 1965). $F_{\mathrm{is}}$, the fixation coefficient of an individual within a subpopulation, shows a loss of heterozygosity in loci excluding *INRA63*, *MAF065*, *McM042*, *McM527*, and *OarFCB20*. The values of the inbreeding coefficient of an individual within the total population, $F_{\mathrm{it}}$, ranged from 0.320 to -0.003. The fixation coefficient of the subpopulation within the whole population, $F_{\mathrm{st}}$, indicates reduction of heterozygosity due to a limit of gene flow and genetic drift among the subpopulation. This result indicates a moderate degree of genetic divergence among subpopulations or breeds of 6.8 % overall. At the same time, the degree of genetic divergence of a subpopulation within the total population amounted to 93.2 %. A similar result was found by Gaouar et al. (2014), ($F_{\mathrm{st}}=6.1$ %), who studied the genetic admixture of North African ovine breeds based on six STR loci (Gaouar et al., 2014). The $F_{\mathrm{st}}$ value was considerably higher than both the $F_{\mathrm{st}}$ values previously reported by Ozerov et al. (2008) and that of Algerian sheep breeds (Gaouar et al., 2015; Abdelkader et al., 2017).

In the present study, we used pairwise population $F_{\mathrm{st}}$ values to provide a measure of the genetic differentiation among sheep breeds (Table 3). The proportion of genetic divergence indicated that moderate differentiation was observed between Kazakh Arkhar-Merino and Edilbay-1 (0.063), Kazakh Arkhar-Merino and Edilbay-2 (0.059), and Kazakh Arkhar-Merino and Kazakh fat-tailed coarse wool (0.057), with a mean of $F_{\mathrm{st}}\;>\;0.05$. These obtained differentiation parameters are generally comparable with $F_{\mathrm{st}}$ values of the fat-tailed Barbarine sheep breed (Sassi-Zaidy et al., 2014). The other investigated sheep population for pairwise genetic differentiation parameters showed values <0.05, which, according to Hartl (1980), indicates a low differentiation among the population. Further, significant genetic differentiation observed ranged from 0.031 to 0.046. Mahmoud et al. (2017) also found significant differentiation between Sawakni, Berberi, and Najdi sheep breeds. However, the lowest $F_{\mathrm{st}}$ coefficient is found between Edilbay-1 and Edilbay-2 since Edilbay-1 and Edilbay-2 are classified as one breed. In order to enhance breed character and to attempt an increase in the status of Edilbay-2, the farmers are buying males from the Edilbay-1 breed, for the reason that the Edilbay-1 breed is a livestock breeding farm with the best quality in Kazakhstan. Consequently, it is clear that migration is going on here. Migration has a great effect on the reduction of genetic differentiation among the population (Laval et al., 2000).

Nei's genetic distances (Nei, 1972) between the five populations of sheep were calculated using 12 STR loci (Table 3), which varied from 0.469 to 0.217. The results of genetic distance of the present study were lower than the findings of Bai et al. (2015), who reported that the genetic distance ranged from 0.21 to 0.62 for Chinese indigenous sheep breeds, which was higher than that of Egyptian sheep breeds (Rushdi et al., 2015). The values of genetic distances showed that the investigated sheep populations are characterized as having a high range of variability of the allele pool, presence or absence of certain alleles, and differences in frequency of occurrence alleles. The largest genetic distance was observed between Kazakh Arkhar-Merino and Edilbay-1 (0.469). These two breeds were absolutely different from each other originally: by phenotype, according to the different branches of stock breeding and geographical places. Kazakh Arkhar-Merino is a meat-woolly breed with fine wool. Kazakh Arkhar-Merino is well adapted to breeding at high altitudes, and these sheep differ favourably from other breeds in conditions of mountain pasture. In contrast, the Edilbay-1 sheep breed is classified as the coarse wool sheep from the meat-fatty category. They are well adapted to the severe desert and semi-desert conditions of Kazakhstan. Also, Kazakh Arkhar-Merino is geographically most distant from Edilbay-1. In comparison with other populations, a closer relationship was found between Kazakh Arkhar-Merino and Kazakh Finewool (0.25), which could be attributed to the low geographical distance between these two populations. The same result has been found between Kermani and Lori-Bakhtiari Pakistani sheep (0.25), which both breed in Iran (Vajed Ebrahimi et al., 2017). Both of them (Kazakh Arkhar-Merino and Kazakh Finewool) refer to the meat and wool types of sheep breeds. Kazakh fat-tailed coarse wool and Edilbay-2 had less similarity to the Kazakh Finewool than Edilbay-1.

Further, to assess the genetic relationships among the population, a phylogenetic tree was constructed using the neighbour joining method (Saitou and Nei, 1987) based on Nei's genetic distance. The results of the phylogenetic tree indicated that the Edilbay-1 and Edilbay-2 sheep breeds were clustered into Kazakh fat-tailed coarse wool, which are coarse wool breeds and have a common origin. However, Kazakh Finewool and Kazakh Arkhar-Merino were grouped in the same node, both being fine wool sheep, as these breeds have the same ancestral background. Likewise, these two breeds were categorized together on the same branch of the phylogenetic tree in the previous study (Ozerov et al., 2008).

In addition, factorial correspondence analysis demonstrated that Kazakh Arkhar-Merino and Kazakh Finewool were isolated from the other studied populations due to the high level of differentiation and no sharing of alleles. Edilbay-2 is an admixture of both Edilbay-1 and Kazakh fat-tailed coarse wool. According to the FCA findings, this is connected with historical origin of these Kazakh sheep breeds.

## 5 Conclusions

In this study, within and among herds, the genetic diversity of five Kazakh sheep populations was assessed using 12 microsatellite markers. Based on our results, all five populations examined show high genetic diversity through a high effective number of alleles, a large mean number of alleles, high PIC values, and 12 completely polymorphic tested microsatellites. Moderate differentiation was found between Kazakh Arkhar-Merino and Edilbay-1, whereas differentiation between Edilbay-1 and Edilbay-2 was lower. Therefore, the evaluation of the results of Nei's genetic distance, neighbour joining, and FCA agreed with the historical origin of animals. As our study showed, although 10 years have passed since the research by Ozerov et al. (2008), it has been discovered that genetic diversity remains in sheep breeds other than Kazakh Arkhar-Merino. The main reasons for this fact are as follows. Scientists and livestock breeders have always been working on the improvement of these breeds. The advantages of this research also include the ability of the breeds to transmit all useful economic traits to their offspring. Breeding work is carried out mainly along the lines using several herds. Of course, not only the best males, but also elite queens, are selected to replenish livestock. Lines of sheep are created according to some outstanding qualities – precocity, weight, size of fat tail, and quality of wool. The work consists mainly of mating animals with distant degrees of kinship in the line. In addition, based on the data obtained, it is possible to recommend the Kazakh Arkhar-Merino breed for the selection against homozygous individuals. Further, the results achieved on STR loci are proposed to be used to control and conserve the genetic diversity of native sheep breeds.

## Data Availability

The materials used the current study are available from the corresponding author on reasonable request.
